# Unique Transcriptomic Profile of Collecting Duct Carcinomas Relative to Upper Tract Urothelial Carcinomas and other Kidney Carcinomas

**DOI:** 10.1038/srep30988

**Published:** 2016-08-03

**Authors:** Gabriel G. Malouf, Eva Compérat, Hui Yao, Roger Mouawad, Veronique Lindner, Nathalie Rioux-leclercq, Virginie Verkarre, Xavier Leroy, Linda Dainese, Marion Classe, Jean-Luc Descotes, Philippe Barthelemy, Mokrane Yacoub, Morgan Rouprêt, Jean-Christophe Bernhard, Chad J. Creighton, Jean-Philippe Spano, Xiaoping Su, David Khayat

**Affiliations:** 1 Department of Medical Oncology, Groupe Hospitalier Pitié-Salpêtrière, University Pierre and Marie Curie (Paris VI), GRC5, ONCOTYPE-Uro, Institut Universitaire de Cancérologie, Assistance-Publique Hôpitaux de Paris, 75013, France; 2 Department of Medical Pathology Groupe Hospitalier Pitié-Salpêtrière, University Pierre and Marie Curie (Paris VI), GRC5, ONCOTYPE-Uro, Institut Universitaire de Cancérologie, Assistance-Publique Hôpitaux de Paris, 75013, France; 3 Department of Bioinformatics and Computational Biology, the University of Texas MD Anderson Cancer Center, Houston, TX, USA; 4 Department of Pathology, CHU Strasbourg, France; 5 Department of Pathology, CHU Rennes, France; 6 Department of Pathology, Hôpital Européen Georges Pompidou, University Paris V, Paris, France; 7 Department of Pathology, CHU Lille, France; 8 Department of Pathology, Trousseau Hospital, Paris, France; 9 Department of Urology, CHU Grenoble, France; 10 Department of Medical Oncology, CHU Strasbourg, France; 11 Department of Pathology, CHU Bordeaux, France; 12 UroCCR French Network for Research on Kidney Cancer (UroCCR), Bordeaux, France; 13 Departments of Medical Urology, Groupe Hospitalier Pitié-Salpêtrière, University Pierre and Marie Curie (Paris VI), GRC5, ONCOTYPE-Uro, Institut Universitaire de Cancérologie, Assistance-Publique Hôpitaux de Paris, 75013, France; 14 Department of Urology, CHU Bordeaux, France; 15 Department of Medicine, Baylor College of Medicine, Houston, TX, 77030, USA

## Abstract

Collecting duct carcinoma (CDC) is a kidney cancer subtype that is thought to arise from principal cells in distal parts of the collecting ducts. Some studies suggested an overlap of CDC with upper tract urothelial carcinoma (UTUC), making the pathological diagnosis challenging. Herein, we performed for the first time transcriptome sequencing of CDC and compared them to UTUC and renal cell carcinoma subtypes. We discovered that CDC displays a unique transcriptomic signature among kidney cancer subtypes, with a putative cell of origin in the distal convoluted tubules. Hierarchical unsupervised clustering reveals that the CDC signature is closer to that of other RCC subtypes than to UTUC, which is similar to that of bladder carcinoma. CDC is characterized by a metabolic shift, with impairment of oxidoreductase activity, pyruvate metabolism and the tricarboxlyic acid cycle, as well as an immunogenic response consistent with increased tumor infiltrating lymphocytes, particularly within metastatic cases. In addition, pathways differentially altered between CDC and UTUC point to a basal-like phenotype of CDC in contrast to the luminal-like signature of UTUC. We conclude that CDC harbors a pathognomonic transcriptomic signature characterized by immunogenic and a metabolic aberrations, indicating that targeting these processes might provide therapeutic options for patients.

Collecting duct carcinoma (CDC) is a rare subtype of renal cell carcinoma (RCC) that is thought to arise from the principal cells in the distal part of the collecting ducts^1^,^2^,^3^. CDC is often considered lethal because it presents in an advanced stage in up to 54% of cases^1^,^4^. Pathology continues to be the gold standard for CDC diagnosis^5^. However, additional histopathological and immunohistochemical examinations (i.e., *GATA3*, *P63*, *PAX8*) may be required for diagnosis as it is sometimes difficult to differentiate CDC from upper tract urothelial carcinoma (UTUC) that originates from the same anatomical region^5^.

In reports of the natural history of CDC, patients with metastatic spread at diagnosis accounted for ~25–50% of all patients, which highlights the aggressiveness of the disease^1^,^4^. However, those reports did not use central pathological reviews that included immunohistochemical staining of selected markers^1^,^4^. The overall survival time of patients with CDC is estimated to be less than one year in the metastatic setting, with poor efficacy of chemotherapy^6^; therefore, there is a need to better characterize these tumors molecularly and identify novel molecular targets. Other RCC subtypes have been extensively analyzed; however, the genetic events that drive the unique behavior of CDC remain unknown.

A study of copy number alterations showed that the genetic profiles differentiated CDC from UTUC^7^. A recent study of 17 CDC cases using targeted sequencing of a panel of 267 genes identified recurrent mutations of *NF2* and *SMARCB1* that were mutually exclusive^8^. Thus, although some progress is being made to decipher the molecular alterations of those tumors, to our knowledge, the transcriptomic profile of CDC has not been reported. This may be related to the rarity of the disease as well as the absence of pathognomonic biomarkers for diagnosing the disease. Among the important unknown factors is whether these tumors display a specific marker that will distinguish them from other RCCs, and from UTUC^5^,^7^,^9^,^10^.

The molecular characterization of kidney tumors has been explored over the past decade, especially the more common subtypes such as clear-cell RCC, papillary RCC, and chromophobe RCC. For rare kidney tumors, data are scarce. Findings indicate that the genes related to kidney cancer, such as *VHL*, *FLCN*, *MET*, *TSC1*, *TSC2*, *SDH* and *FH*, are involved in metabolic pathways related to oxygen and iron or nutrient sensing, which thus characterizes kidney cancer as a disease of cell metabolism^11^. Whether CDC is also a metabolic disease remains unknown.

To chart the transcriptomic profile of CDC, we took advantage of CDC cases collected over the last decade in several academic institutions in France. Our analysis identified a unique transcriptomic signature of CDC that is driven by the deregulation of metabolic and immunogenic genes. We further defined its putative cell of origin, which we found to be consistent with the distal convoluted tubules (DCTs) of the kidney.

## Results

### Clinical features

Overall, 17 patients with pathological diagnosis of CDC were identified (Table 1). The median age was 60 years (range: 17–78 years), which was similar to that for classical clear-cell RCC. Male patients predominated, with a ratio of male/female of ~2:1. All the patients except two had undergone a radical nephrectomy, and all but one had stage III-IV disease, with 44% of the patients presenting at diagnosis with distant metastasis. The 9 primary muscle-invasive UTUC cases selected as a control were classified as stage III-IV and stage II in 77.8% and 22.2% of the respective cases.

### Unique transcriptomic signature of CDC

**Figure 1:**
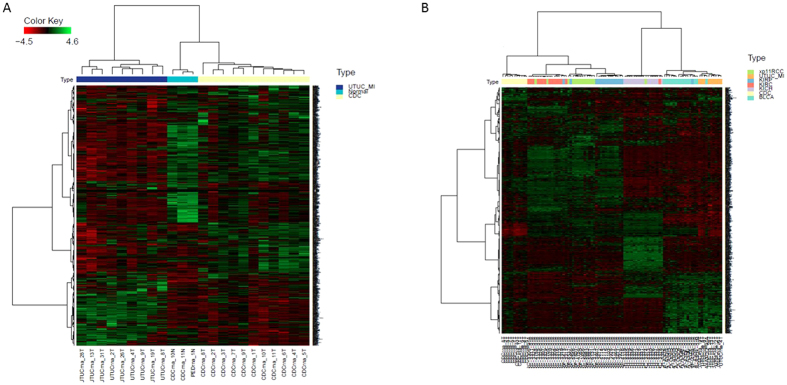
Unsupervised clustering for the most differentially expressed mRNA. (**A**) CDC forms a unique cluster compared to muscle-invasive UTUC and normal kidney tissue. (**B**) CDC compose a unique cluster compared to urothelial tumor (bladder carcinoma and UTUC) and other renal cell carcinoma subtypes, clear-cell RCC (KIRC), papillary RCC (KIRP), translocation RCC (Xp11RCC) and chromophobe RCC (KICH).

To assess whether CDC displays a unique signature compared to UTUC, we performed unsupervised hierarchical clustering of CDC, UTUC and normal kidney samples. All CDC cases clustered together compared to UTUC, which also formed a distinct cluster (Fig. 1A). Of note, normal kidney samples were closer to CDCs than to UTUCs, pointing to the higher similarity of the CDC transcriptome to that of normal kidney tissue than to normal urothelium. Those results were confirmed using principal component analysis (Supplementary Figure 1).

To clarify whether the CDC transcriptome is unique compared to those of other RCC subtypes and urothelial carcinomas, we performed hierarchical unsupervised clustering using several subtypes of RCC (clear-cell RCC, papillary RCC, chromophobe and translocation RCC), bladder carcinomas and UTUC (Fig. 1B). Strikingly, we found that UTUC cases clustered with bladder carcinomas, indicating similar transcriptomic profiles. Conversely, CDC clustered with other RCC subtypes within another distinct cluster. Thus, we conclude that CDCs represent a unique cluster among RCC subtypes, which points to a pathognomonic signature for this disease.

### Nephron site of origin of CDC

**Figure 2:**
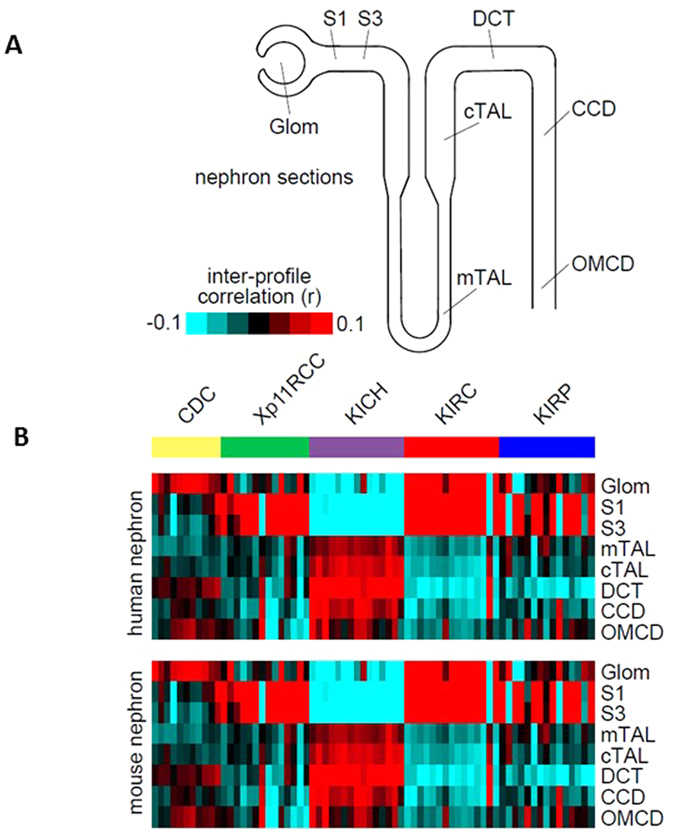
Association of CDC expression patterns with those of specific regions of the nephron. (**A**) The 8 kidney nephron regions evaluated: Glom, glomerulus; S1 and S3, the proximal tubule; mTAL, medullary thick ascending limb of Henle’s loop; cTAL, cortical thick ascending limb of Henle’s loop; DCT, distal convoluted tubule; CCD, cortical collecting duct; OMCD, outer medullary collecting duct. (**B**) Heat maps showing inter-sample correlations (red, positive) between mRNA profiles of RCC (columns) and mRNA profiles of nephron anatomical sites (rows). CDC, collecting duct carcinomas; KIRC, TCGA clear-cell RCC cases; KIRP, TCGA papillary RCC cases; Xp11RCC, TCGA translocation RCC cases; KICH, TCGA chromophobe RCC cases.

On the basis of immunohistochemical observations of the similarity of RCC subtypes, it has been postulated that CDC arises from cells in the distal part of the collecting ducts. However, this assumption has not been confirmed by genome-wide molecular characterizations. Thus, we examined our gene expression data in the context of an external gene expression dataset of normal kidney cells microdissected from eight distinct regions of the nephron^12^. Through supervised analysis, we globally compared the gene expression data of the CDC samples and each TCGA type (clear-cell RCC, chromophobe RCC, papillary RCC and Xp11 RCC) with that of each cell type in the nephron structure, and found high mRNA expression correlations for CDC with the DCTs (Fig. 2). Thus, our findings are consistent with CDC originating from the DCTs, which differentiates CDC from other kidney cancers (clear-cell RCC, papillary RCC) that have proximal origins in the nephron. Another important distinction is that our findings did not show high similarity of gene expression between CDC and the thick ascending limb of Henle’s loop; whereas the gene expression of chromophobe RCC was highly similar to that of the cells of the distal nephrons.

### CDC is a metabolic disease

**Figure 3:**
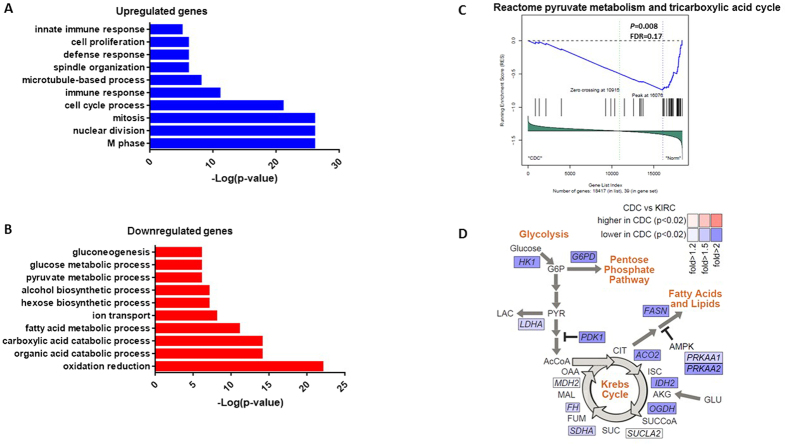
(**A**,**B**) Functional enrichment analysis of the genes differentially expressed between CDC and normal kidney tissue; (**A**) overexpressed in CDC; (**B**) underexpressed in CDC. (**C**) Gene set enrichment analysis showing impairment of pyruvate metabolism and tricarboxylic acid cycle in CDC. (**D**) Schematic of differential expression patterns of CDC versus clear-cell RCC in metabolism-related pathways, with a focus on gene expression patterns previously associated with Warburg-like effects in kidney cancer. P-values calculated by a t-test.

We then compared CDC gene expression to that of normal kidney tissues. Overall, 308 genes (1.7%) were upregulated ([FC] ≥ 2 and FDR < 0.05). Gene ontology analysis using DAVID revealed that the highly enriched genes were related to the M phase of the mitotic cell cycle (p = 1.12 × 10^−25^) and to immune response (p = 4.8 × 10^−11^) (Fig. 3A; Supplementary Table 2). Conversely, the 574 genes (3.1%) that were downregulated (FC ≤ −2 and FDR < 0.05) were related to oxidation reduction (p = 1.69 × 10^−22^) as well as fatty acid (p = 4.1 × 10^−11^), glucose (p = 3.3 × 10^−6^) and pyruvate (p = 3.3 × 10^−6^) metabolic processes (Fig. 3B; Supplementary Table 3). This was confirmed using gene set enrichment analysis (GSEA), which revealed impairment of oxidoreductase activity (p = 0.01, FDR = 0.16), pyruvate metabolism, and the tricarboxylic acid (TCA) cycle (Fig. 3C), as well as aerobic respiration (Supplementary Figure 2). Of note, the *AMPK* gene was downregulated by at least 6.25 fold in CDC versus normal kidney tissue (p = 0.0004; FDR = 0.01). We then assessed whether the metabolic shift observed in CDC is more important than that observed in clear-cell RCC. Our comparison with normal kidney tissue showed that CDC harbored an impaired TCA cycle compared to that associated with clear-cell RCC (Fig. 3D). We observed similar findings when comparing CDC with papillary RCC (data not shown).

### CDC is an immunogenic RCC subtype

**Figure 4:**
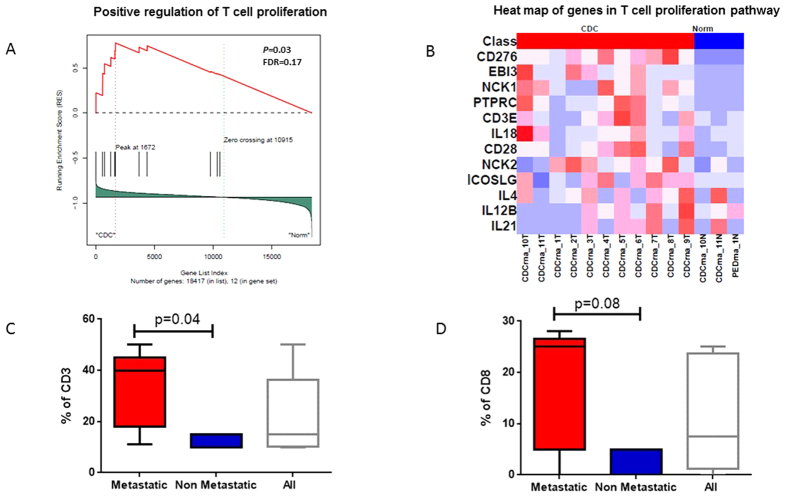
CDC and the immune system. (**A**,**B**) Gene set enrichment analysis showing enrichment for positive regulation of T cell proliferation pathways, with the corresponding heatmap of differentially expressed genes. (**C**) Percentage of CD3 tumor infiltrating lymphocytes in all CDC cases and in metastatic versus non-metastatic cases. (**D**) Percentage of tumor infiltrating CD8 lymphocytes in all CDC cases and in metastatic versus non-metastatic cases. P-values calculated by a t-test.

As genes of the immune response were overexpressed in CDC compared with their expression in normal kidney tissue, we used GSEA to clarify which immune pathways were deregulated. As a result, we discovered several pathways, including the early activation of T lymphocytes (p = 0.007, FDR = 0.05), positive regulation of lymphocyte activation (p = 0.01, FDR = 0.14), positive regulation of the immune system process (p = 0.04; FDR = 0.12) and positive regulation of T cell proliferation (p = 0.03; FDR = 0.17) (Fig. 4A,B). We then sought to identify whether the tumor tissue in CDC was globally infiltrated by T lymphocytes, which would be consistent with aberrant T cell infiltration in this setting. We found that the overall median tumor infiltrating lymphocytes (TIL) percentage in CDC assessed using CD3 staining was 22% (range: 0–50%), with a higher statistically significant percentage in metastatic versus non-metastatic tumors (p = 0.04) (Fig. 4C). Similarly, the median CD8 TIL percentage was 11% (range: 0–25%), with a trend toward a higher percentage in metastatic versus non-metastatic tumors (p = 0.08) (Fig. 4D).

### Analysis of genes differentially expressed between CDC and UTUC

**Figure 5:**
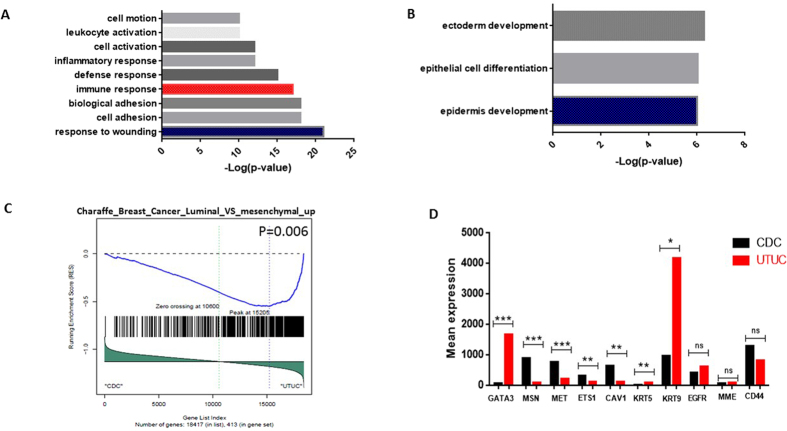
(**A**,**B**) Functional enrichment analysis of the genes differentially expressed between CDC and UTUC; (**A**) overexpressed in CDC; (**B**) underexpressed in CDC. (**C**) Gene set enrichment analysis showing enrichment of luminal-like breast cancer signature versus mesenchymal signature in CDC compared to UTUC. (**D**) Box-plots of the expression of 10 genes previously reported as differentially expressed between luminal-like and basal-like breast cancer tumors.

As the differential diagnosis of CDC versus UTUC may pose a challenge, we looked at the genes that were significantly differentially expressed between them (fold change ≥2 or ≤−2 and FDR < 0.05), with the aim of discovering clinical biomarkers. Overall, 1,886 and 823 genes out of 18,417 were overexpressed and downregulated, respectively. Gene ontology using DAVID pathway analysis revealed that genes overexpressed in CDC were highly enriched for wound healing and the activation of both leukocytes and the immune system (Fig. 5A). In contrast, the genes that were enriched and downregulated in CDC were related to ectoderm differentiation and the epidermis (Fig. 5B; Supplementary Table 4), which indicates that those genes define the cell’s identity.

To confirm these findings, we used GSEA, which showed enrichment for gene sets related to the positive regulation of T cells (p = 0.02) and leukocyte activation (p = 0.02). Importantly, we also discovered enrichment of the genes in the basal-like breast cancer signature in CDC compared to the enrichment of the luminal-like signature in UTUC (p = 0.005) (Fig. 5C). In particular, the expression of 7 out of 10 genes (70%) previously demonstrated to discriminate between the luminal-like and basal-like signatures of breast cancer were differentially expressed between CDC and UTUC^13^ (Fig. 5D; Supplementary Table 5). Those genes are *CAV1*, *CD44*, *EGFR*, *MET*, *ETS1*, *GATA3*, luminal cytokeratin *CK19*, basal cytokeratin *CK5/6*, *CD10* and the *ERM* protein moesin^13^. We found this to be of interest because *GATA3* is used in the clinic for the differential diagnosis of UTUC versus CDC.

### Identification of biomarkers for genes differentially expressed between CDC and UTUC

To discover new markers that may serve to differentiate between UTUC and CDC, we investigated genes that showed the most differential expression. Among the top downregulated genes in CDC, we identified *GATA3*, *TP63*, *KRT17*, *KRT7*, *KRT20* as well as *UPK2*, *UPK1A and UPK3A* (Supplementary Table 6). Our finding of *GATA3* and *TP63* being downregulated is consistent with these genes being urothelial markers. All our CDC cases with available material showed negative staining for *GATA3* and *TP63*. Among the top overexpressed genes in CDC, we identified *CDH6* and *POU3F3*, which are involved in kidney development (Supplementary Table 7). The *CRYAB* gene, which has been shown to be highly expressed in basal-like breast tumors with poor prognoses, was the top overexpressed gene in CDC that differentiated it from UTUC^14^.

## Discussion

This is the first study to investigate the transcriptomic profile of CDC and compare it to that of other RCC subtypes as well as urothelial carcinomas. Our data shed some important insights on understanding CDC. First, we demonstrate that CDC is molecularly distinct from other RCC subtypes, with a unique gene expression signature. In addition, contrary to putative origins in the cells of the distal nephrons^1^,^2^,^3^, our data suggest that CDC might originates from the DCT of the nephron, which is the segment between the proximal tubules and the distal part of the nephron. Histologically, the cells that form the DCT are located in the vicinity of glomeruli, which might explain the similarity between CDC and the glomeruli expression profils; however, this correlation was less important than that with the cells of the DCT. The study by Cheval *et al*., which was the first to document the architecture of microdissected nephron cells, determined that the cells of the DCT are transcriptionally closer to those of the glomeruli than to cells of the distal nephrons, which is consistent with our observations^15^.

Second, our data provide the first indication that CDC is a metabolic disease, which also characterizes other kidney tumors^11^. Indeed, gene expression analysis unraveled profound impairment of oxidoreductase in CDC, with increased aerobic glycolysis and decreased expression of the *AMPK* gene, which is also a feature of aggressive clear-cell RCC and papillary RCC^16^,^17^. The mechanism behind the metabolic effects of CDC needs to be clarified. In other RCC subtypes, mutations of *SDHB* or *FH* have been reported to be consistent with this phenotype^11^. Pal *et al*. found only 2 of 9 CDC cases to harbor homozygous *FH* loss; they observed no *FH* or *SDHB* mutations^8^. Further studies using next-generation sequencing are needed to discover whether the development of this disease is linked to mutations in genes related to metabolism. This observation might have important implications because it extends the paradigm of kidney cancer as a metabolic disease that is largely espoused by the Linehan group^18^. However, determining the extent to which metabolic alterations in this disease are similar to those in other RCC subtypes necessitates future research efforts.

Third, to help identify biomarkers that may serve to differentially diagnose CDC versus UTUC, we analyzed the significant differential signature that defined the origin of the respective tumor cells. We confirmed the usefulness of the previously described markers *GATA3* and *P63*, and discovered novel markers such as *UPK* genes that are potentially useful for a differential diagnosis. We conducted unbiased observations in contrast to previous studies that used a single-gene approach^9^. These data add another level of evidence to the distinction between CDC and UTUC in addition to a genetic study using copy number variation^7^. We also identified a mesenchymal signature of CDC that is consistent with the basal-like breast cancer signature^13^. However, regarding the small number of cases of CDC and UTUC assessed, it needs to be clarified in a larger dataset whether the distinction between basal-like and luminal-like features will remain. Indeed, when we extrapolate the data from bladder cancer, which can express, basal-like, luminal-like and p-53 like subtypes, to UTUC, we do not know if in a larger dataset of UTUC a subgroup will show also a basal-like feature as compared to CDC. Thus, we speculate that the mechanisms behind tumor aggressiveness in this disease might use epithelial-mesenchymal transition as a driving force of cellular plasticity that favors invasion and metastasis.

Last, our analysis revealed a high enrichment of the immune signature in CDC. Examination of tumor slides for immune infiltration using CD3 and CD8 staining confirmed a high percentage of T cell infiltrates in CDC. Importantly, the CD3 infiltration percentage was higher in metastatic tumors than in non-metastatic tumors; a similar trend was observed for CD8 cells. Increased concentration of CD8 lymphocytes has been associated with good outcomes in colon and breast cancers^19^; however, a recent study identified a high percentage of CD8 cells in clear-cell RCC as a poor prognostic marker^20^. A possible explanation is that those lymphocytes are inactive^20^. The small number of cases does not allow for a definitive conclusion to be drawn from our study; additional studies are needed to clarify the nature of those immune cells. In the era of immune checkpoint inhibitors and for a disease with limited benefit from targeted agents, our data suggest that immunotherapy may be feasible for CDC.

The strength of our study is the collection of frozen material within a 10-year period in several institutions in France, combined with a central pathological review and the use of UTUC samples as the control. The limitations of our study are the small number of cases and the absence of an independent validation dataset.

We conclude that CDC is a unique subtype of RCC with a distinct transcriptomic profile closer to that of kidney tumors than to UTUC. CDC may originate in the DCT cells of the kidneys. We also demonstrate that CDC is an immunogenic disease with a high level of immune lymphocyte infiltrates. In addition, CDC is a metabolic disease similar to other RCC subtypes, which expands the paradigm of kidney cancer as a metabolic disease.

### Patients and Methods

#### Case collection

Patient samples with confirmed diagnosis of CDC were collected from different pathology departments in France after local committee approval. To differentiate CDC from UTUC, only cases of CDC for which a confirmed pathological diagnosis was performed on the primary tumor were selected. All cases were extensively reviewed by one expert pathologist (E.C.), who confirmed the diagnosis using recently published criteria^21^. When necessary, immunohistochemistry for selected markers was performed (i.e., *GATA3* and *P63*). All but one patient had undergone primary nephrectomy, which allowed for the pathological diagnosis. To identify biomarkers that may be used to differentiate between the diagnosis of CDC and UTUC, we selected 9 muscle-invasive UTUC cases from the Pitie-Salpetriere Hospital, including 7 cases classified as pT3-4 and 2 cases classified as pT2. Four of those 9 (44.4%) cases showed lymph node involvement. Two (22.2%) of them had metastatic disease at presentation.

All patients had previously provided written inform consent for tumor collection and analysis. Clinical and pathologic data were collected in each participating institution by pathologists and/or urologist and medical oncologists. The study was approved by the ethical committee of the Pitie-Salpetriere Hospital (IDF-6, Ile de France). The collection and use of tissues followed procedures that are in accordance with the ethical standards formulated in the Declaration of Helsinki.

### Histopathologic and immunohistochemical analysis of immune infiltrate

Tissue microarrays were constructed for the 12 CDC cases for which material was available using 6 cores to represent different areas of the tumors. All samples were stained and analyzed for CD3 and CD8 cells using standard routine procedures. The tumor infiltrating lymphocyte (TIL) percentage was evaluated by an expert pathologist (E.C.) as the percentage of lymphocytes present in all the areas assessed (tumor and stroma) according to CD3 and CD8 immunostaining. Comparisons between TIL percentages were performed with a nonparametric t-test using GraphPad Prism ®.

### RNA sequencing

RNA extraction was performed on CDC, UTUC, and normal kidney tissue samples using the RNeasy Kit (Qiagen) according to the manufacturer’s instructions. Eleven of the CDC cases met the criteria for RNA sequencing. As a control, we selected 3 adjacent kidney tissue samples (n = 3) and 9 UTUC samples. Normal kidney tissues were taken from normal kidney areas located at least 2 centimeter distance from the primary tumors and were checked by our pathologist (E.C.) for the absence of metastatic cells. After controlling for the quality of the initial samples, rRNA depletion was performed for the total RNA for each sample, followed by random-primed and stranded cDNA preparation and quality control. Total RNA was converted into a library of template molecules for sequencing on Illumina HiSeq2000, with a paired-end read length of 100–125 nt. The sequencing depth for each case is reported in Supplementary Table 1. We compared these cases with kidney tumor samples obtained from The Cancer Genome Atlas (TCGA), which are also identified in Supplementary Table 1.

### RNA sequencing mapping and analysis

The raw reads in FASTQ format were aligned to the reference human genome, hg19, using MOSAIK alignment software. MOSAIK implements the Smith-Waterman algorithm to perform gap alignment. The overlaps between the aligned reads and annotated genes were counted using HTSeq software (http://www-huber.embl.de/users/anders/HTSeq/doc/overview.html). The gene counts were normalized using the scaling factor method. If the number of overlapping reads of any given gene was less than one per million total mapped read for all samples, that gene was excluded from further analysis. A hierarchical clustering analysis was performed using the Pearson correlation coefficient as the distance metrics and Ward’s linkage rule. Principal component analysis was also applied to discover the multi-gene structure. A negative binomial generalized linear model was fit to each gene expression with the sample tissue type (CDC vs. muscle-invasive UTUC vs. normal kidney) as the covariate. Then Wald’s test was applied to determine whether there was any difference in the expression of a gene between any two types of tissue. The Benjamini-Hochberg method was used to control the false discovery rate (FDR).

Another control was a dataset randomly selected from TCGA, which encompassed clear-cell RCC (n = 15), papillary RCC (n = 15), translocation RCC (n = 15) and bladder carcinoma (n = 15). The samples evaluated are listed in Supplementary Table 1. Hierarchical unsupervised clustering was used to discover whether the transcriptomic profile of CDC is similar to that of other kidney tumors or UTUC.

The Cheval *et al*. dataset^12^ of gene expression profiles from various sites of the nephron (both human and mouse tissues) was analyzed as previously described^22^. For each gene in the RCC dataset (combined CDC, KIRC, KICH and KIRP), expression values were centered across tumors on the mean centroid of the three TCGA projects; within each of the human and mouse datasets from the Cheval nephron atlas, values were centered on the median across samples. Using the centered datasets, for each RCC and Cheval mRNA profile, we computed the global inter-profile correlation (by Pearson’s), using all ~4000 genes in common (most genes in the Cheval dataset being represented in the RCC dataset).

## Additional Information

**How to cite this article**: Malouf, G. G. *et al*. Unique Transcriptomic Profile of Collecting Duct Carcinomas Relative to Upper Tract Urothelial Carcinomas and other Kidney Carcinomas. *Sci. Rep.* **6**, 30988; doi: 10.1038/srep30988 (2016).

## Supplementary Material

Supplementary Information

Supplementary Dataset 1

## Figures and Tables

**Table 1:** Clinicopathological features of 17 cases of collecting duct carcinoma.

ID of CDC case	Age (years)	Sex	Surgery	Tumor size (cm)	pTMN	AJCC stage	Metastatic site(s)	Current status	RNAseq	IHC
BEL-1T	46	F	nephrectomy	14	pT3bN2M0	3		Died (12 months)	Yes	No
BEL-2T	17	M	nephrectomy	5.5	pT3aN2M0	3		NED	Yes	Yes
BEL-3T	42	F	nephrectomy	4.5	pT3aNxM0	3		NED	Yes	Yes
BEL-4T	65	M	nephrectomy	7.5	pT3aN2M1	4	lung	Died (13 months)	Yes	Yes
BEL-5T	56	M	partial nephrectomy	5	PT3aNxM0	3		AWD	Yes	Yes
BEL-6T	49	M	nephrectomy	9	pT3aN1M0	3		AWD	Yes	Yes
BEL-7T	28	F	nephrectomy	9	pT3aN2M1	4	paravertebral muscle	Died (26 months)	Yes	No
BEL-8T	64	M	nephrectomy	3	pT4bN1M1	4	liver, adrenal gland	AWD	Yes	No
BEL-9T	67	M	nephrectomy	8.7	pT3bN1M1	4	bone	Died (10 months)	Yes	Yes
BEL-10T	60	F	nephrectomy	6.5	pT3N2M0	3		AWD	Yes	Yes
BEL-11T	63	M	nephrectomy	7	pT2NxM0	2		NED	Yes	No
BEL-12T	73	M	nephrectomy	11	pT3bNxM1	4	liver and lung	Died (9 months)	No	Yes
BEL-13T	71	M	nephrectomy	9	T3aN0M0	3		AWD	No	Yes
BEL-14T	63	M	nephrectomy	8	T3aNxM1	4	lung and bone	AWD	No	No
BEL-17T	78	F	nephrectomy	6	T3N2M0	3		AWD	No	Yes
BEL-18T	33	F	No	8	T3bN2M1	4	lung, liver and bone	Died (11 months)	No	Yes
BEL-19T	59	M	nephrectomy	8	T3bN2M1	4	bone, lung	AWD	No	Yes

Note: pTMN, pathologic tumor stage; AJCC stage, American Joint Committee on Cancer stage; NED, no evidence of disease; AWD, alive with disease; IHC.
