# Supplementary material for: Unique Transcriptomic Profile of Collecting Duct Carcinomas Relative to Upper Tract Urothelial Carcinomas and other Kidney Carcinomas
Source: Sci Rep. 2016 Aug 3;6:30988. doi: 10.1038/srep30988 (PMC4971524; doi:10.1038/srep30988)
Supplement: Supplementary Information [file srep30988-s1.pdf]

Unique Transcriptomic Profile of Collecting Duct Carcinomas Relative to Upper Tract Urothelial Carcinomas and other Kidney Carcinomas

Gabriel G. Malouf[1*], Eva Compérat[2*], Hui Yao[3], Roger Mouawad[1], Veronique Lindner[4] Nathalie Rioux-leclercq[5], Virginie Verkarre[6], Xavier Leroy[7], Linda Dainese[8], Marion Classe[1], Jean-Luc Descotes[9], Philippe Barthelemy[10], Mokrane Yacoub[11,12], Morgan Rouprêt[13], Jean-Christophe Bernhard[12,14], Chad J. Creighton[15], Jean-Philippe Spano[1], Xiaoping Su[3*], David Khayat[1*]

[1]Departments of Medical Oncology, [2]Pathology, [13]Urology, Groupe Hospitalier Pitié-Salpêtrière, University Pierre and Marie Curie (Paris VI), GRC5, ONCOTYPE-Uro, Institut Universitaire de Cancérologie, Assistance-Publique Hôpitaux de Paris, 75013, France. [3]Department of Bioinformatics and Computational Biology, the University of Texas MD Anderson Cancer Center, Houston, TX, USA. [4]Department of Pathology, CHU Strasbourg, France. [5]Department of Pathology, CHU Rennes, France. [6]Department of Pathology, Hôpital Européen Georges Pompidou, University Paris V, Paris, France. [7]Department of Pathology, CHU Lille, France. [8]Department of Pathology, Trousseau Hospital, Paris, France. [9]Department of Urology, CHU Grenoble, France. [10]Department of Medical Oncology, CHU Strasbourg, France. [11]Department of Pathology, CHU Bordeaux, France. [12]UroCCR French Network for Research on Kidney Cancer (UroCCR), Bordeaux, France. [14]Department of Urology, CHU Bordeaux, France. [15] Department of Medicine, Baylor College of Medicine, Houston, TX 77030, USA

*These authors are co-senior authors

Supplementary Figure Legends

Supplementary Figure 1. Principal component analysis showing that CDC is different from UTUC and normal kidney tissue.

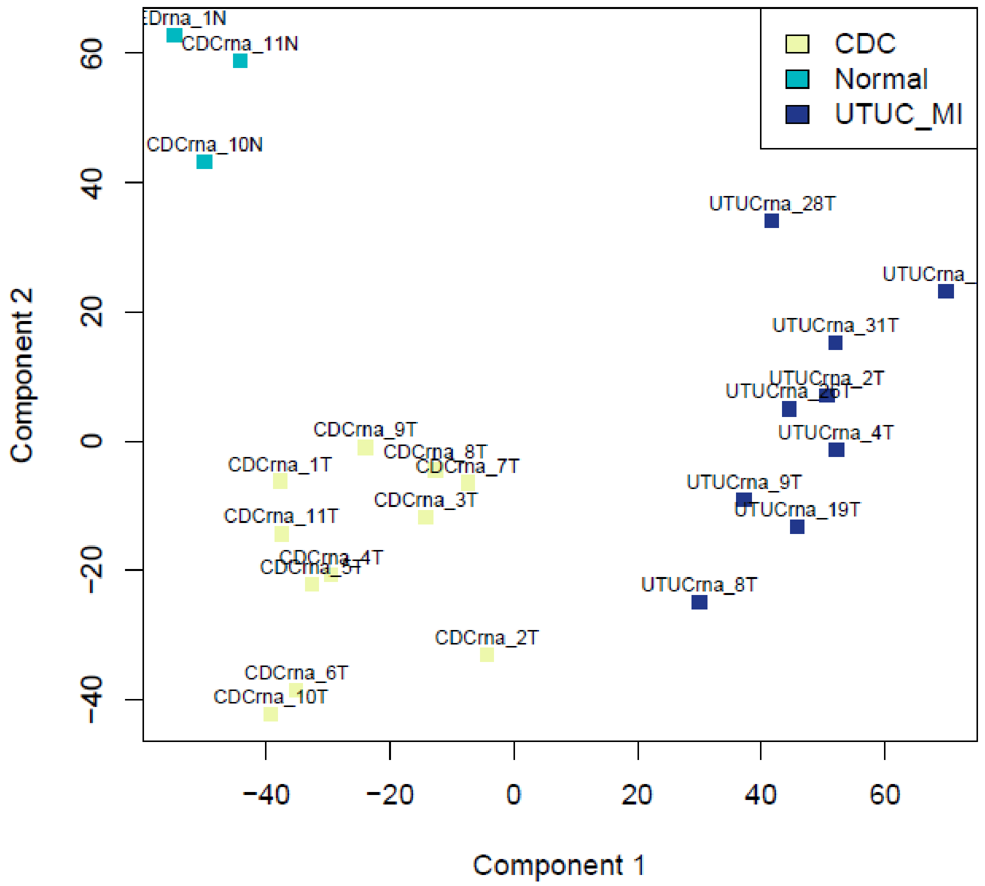

CDC
Normal
UTUC_MI
Component 1
Component 2
Drna_1N
CDCrna_11N
CDCrna_10N
UTUCrna_28T
UTUCrna_
UTUCrna_31T
UTUCrna_2T
UTUCrna_4T
UTUCrna_9T
UTUCrna_19T
UTUCrna_8T
CDCrna_9T
CDCrna_8T
CDCrna_7T
CDCrna_1T
CDCrna_3T
CDCrna_11T
CDCrna_4T
CDCrna_2T
CDCrna_6T
CDCrna_10T

Supplementary Figure Legends

Supplementary Figure 2. Gene set enrichment analysis showing enrichment of aerobic glycolysis in CDC compared to UTUC.

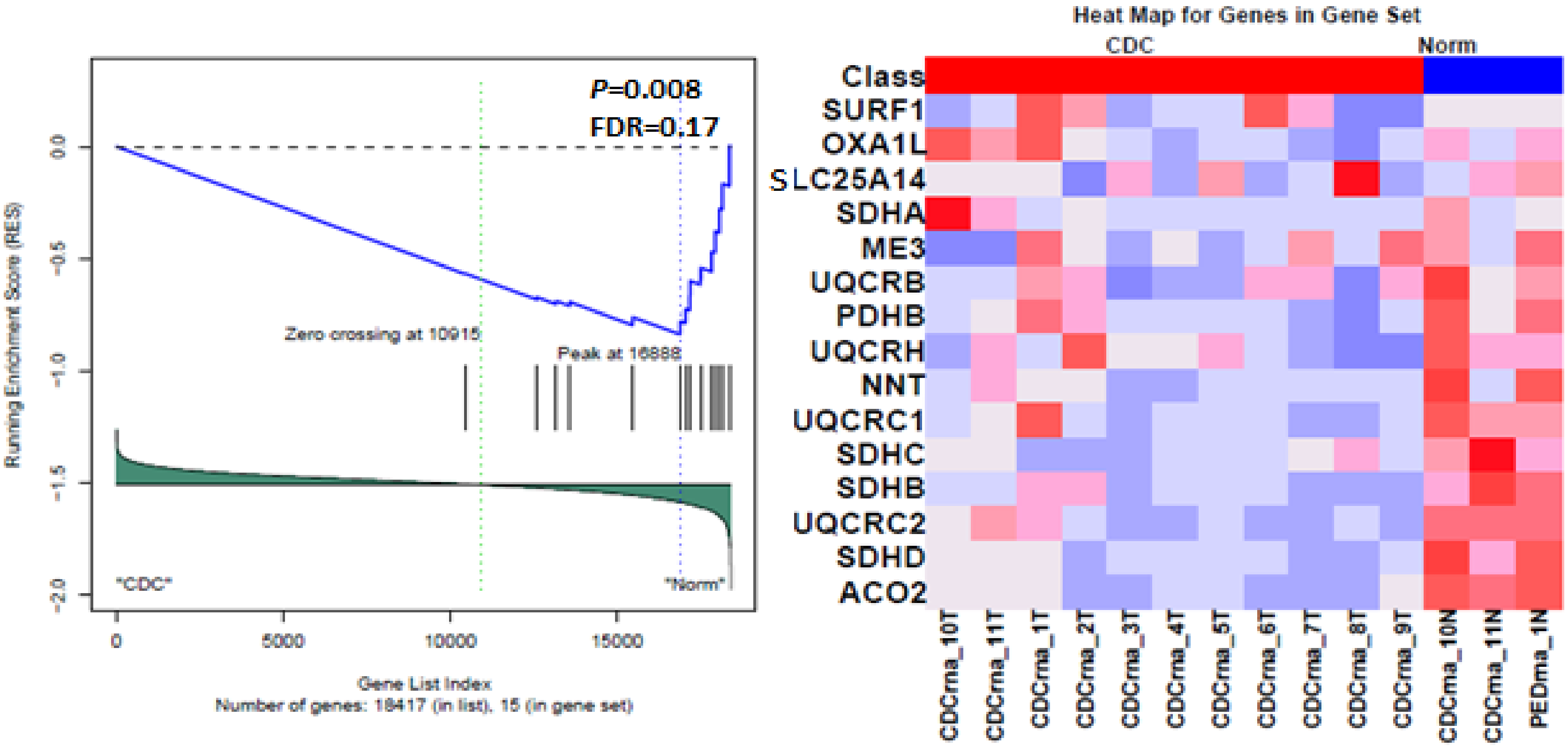

P=0.008
FDR=0.17
Running Enrichment Score (RES)
0.0
-0.5
-1.0
-1.5
-2.0
Zero crossing at 10915
Peak at 16888
"CDC"
"Norm"
0
5000
10000
15000
Gene List Index
Number of genes: 18417 (in list), 15 (in gene set)
Heat Map for Genes in Gene Set
CDC
Norm
Class
SURF1
OXA1L
SLC25A14
SDHA
ME3
UQCRB
PDHB
UQCRH
NNT
UQCRC1
SDHC
SDHB
UQCRC2
SDHD
ACO2
CDCma_10T
CDCma_11T
CDCma_1T
CDCma_2T
CDCma_3T
CDCma_4T
CDCma_5T
CDCma_6T
CDCma_7T
CDCma_8T
CDCma_9T
CDCma_10N
CDCma_11N
PEDma_1N
